# BMC *Ophthalmology* reviewer acknowledgement 2014

**DOI:** 10.1186/1471-2415-15-4

**Published:** 2015-01-30

**Authors:** Catherine M Rice, Diana M Marshall

**Affiliations:** BioMed Central, Floor 6, 236 Gray’s Inn Road, London, WC1X 8HB UK

## Abstract

The editors of BMC Ophthalmology would like to thank all our reviewers who have contributed to the journal in Volume 14 (2014).

**Osama Ababneh**

Jordan

**Mohammed Abdull**

UK

**Marwan Abouammoh**

Saudi Arabia

**Grazyna Adamus**

USA

**Aniruddha Agarwal**

USA

**Ashish Ahuja**

USA

**Tan Aik Kah**

Malaysia

**Hideo Akiyama**

Japan

**Luciana Alencar**

Brazil

**Christiane Al-Haddad**

Lebanon

**Aliaa Ali**

Egypt

**Mohammad Ali**

India

**Majed Alkharashi**

Saudi Arabia

**Fowzan Alkuraya**

Saudi Arabia

**Riham Allam**

Egypt

**Winfried Amoaku**

UK

**Marcus Ang**

Singapore

**Nicola Anstice**

New Zealand

**Florent Aptel**

France

**Ilir Arapi**

Italy

**Samuel Arba Mosquera**

Spain

**Luis Arias**

Spain

**Paul Artes**

Canada

**Maria Angeles Asencio**

Spain

**Salman Asghar**

Pakistan

**Ioannis Aslanis Aslanides**

Greece

**Sam Ebenezer Athikarisamy**

Australia

**Francesco Attanasio**

Italy

**Venkata Avadhanam**

UK

**Abdulkabir Ayanniyi**

Nigeria

**Ramesh Ayyala**

USA

**Simon Bababeygy**

USA

**Simon Backhouse**

Australia

**Sehyun Baek**

Korea, South

**Berker Bakbak**

Turkey

**Konstantinos Balaskas**

UK

**Mehmet Balci**

Turkey

**Melike Balikoglu-Yilmaz**

Turkey

**Touka Banaee**

Iran

**Katayoon Baradaran Ebrahimi**

USA

**Piero Barboni**

Italy

**John Barbur**

UK

**Brendan Barrett**

UK

**John Barry**

UK

**Peter Barry**

Ireland

**Robert Barry**

UK

**Ken Bassett**

Canada

**Roberto Bellucci**

Italy

**Antonio Benito**

Spain

**Kanchan Bhan**

UK

**Alexandre Bisdorff**

Luxembourg

**Paul Bishop**

UK

**Michael Boland**

USA

**Beatrice Bourghardt Peebo**

Sweden

**Mete Bozkurt**

Turkey

**Beau Bruce**

USA

**Paolo Brusini**

Italy

**Franziska Bucher**

Germany

**Ibrahim Buttanri**

Turkey

**Halil Çagatay**

Turkey

**Carlo Cagini**

Italy

**Jill Carlton**

UK

**Zia Carrim**

UK

**Marissa Carter**

USA

**Robert Casson**

Australia

**Ingele Casteels**

Belgium

**Heidi Cate**

UK

**Cestmir Cejka**

Czech Republic

**Mario Chacon**

Mexico

**Clara Chan**

Canada

**Errol Chan**

Singapore

**William Charman**

UK

**Tanveer Chaudhry**

Pakistan

**Sunita Chaurasia**

India

**Angela Chen**

USA

**Ching-Shih Chen**

USA

**Katherine Chen**

USA

**Yihe Chen**

USA

**Clarissa Cheng**

Singapore

**Andreea Chih**

USA

**Pauline Cho Hong**

Kong

**Hyuk Jin Choi**

Korea, South

**Kup-Sze Choi**

Hong Kong

**Kam-Lung, Kelvin Chong**

Hong Kong

**Sung Kun Chung**

Korea, South

**Nerida Cole**

Australia

**Miriam Conway**

UK

**Jessica Cooke Bailey**

USA

**Ciro Costagliola**

Italy

**John Crews**

USA

**Valente Cristiana**

Italy

**Phillippa Cumberland**

UK

**Mary Daly**

USA

**Erika Damato**

UK

**Vivek Dave**

India

**Annelies De Klein**

Netherlands

**Gabor Deak**

Austria

**Dawn Decarlo**

USA

**Alastair Denniston**

UK

**Alexandre Denoyer**

France

**Manishi Desai**

USA

**Vasilios Diakonis**

Greece

**Andrew Dick**

UK

**Xiaoyan Ding**

China

**Ali Djalilian**

USA

**Mangat Ram Dogra**

India

**Thomas Dohlman**

USA

**Kimberly Drenser**

USA

**Chiara Eandi**

Italy

**Deepak Edward**

Saudi Arabia

**Nathan Efron**

Australia

**Allen Eghrari**

USA

**Alvin Eisner**

USA

**Tom Eke**

UK

**Bengu Ekinci Koktekir**

Turkey

**Mohamed El Sanharawi**

France

**Yamine El Sayed**

Egypt

**Mostafa Elgohary**

UK

**Sitki Ermis**

Turkey

**Marie-Helene Errera**

France

**Richard Fairless**

Germany

**Miguel Faria-Ribeiro**

Portugal

**Sepehr Feizi**

Iran

**Yifan Feng**

China

**Maryam Ferdousi**

UK

**Roberto Fernandez Buenaga**

Spain

**Antonio Ferreras**

Spain

**Irene Floriani**

Italy

**Paolo Fogagnolo**

Italy

**Angie Fong**

Hong Kong

**Alex Fonollosa Calduch**

Spain

**Gary Foster**

USA

**Nikitas Fountoulakis**

Greece

**Ian Francis**

Australia

**Sandra Franco**

Portugal

**Jeffrey Freedman**

USA

**Amanda French**

Australia

**Paolo Frezzotti**

Italy

**Fusako Fusimura**

Japan

**Jeremias Galletti**

Argentina

**Juan Eduardo Gallo**

Argentina

**Alfredo Garcia-Layana**

Spain

**Sunir Garg**

USA

**Zisis Gatzioufas**

Switzerland

**Jian Ge**

China

**Ronald Gentile**

USA

**Ilias Georgalas**

Greece

**Joann Giaconi**

USA

**Andrea Giani**

Italy

**Jonathan Gibson**

UK

**Fleur Goezinne**

Netherlands

**E-Shawn Goh**

Singapore

**Gokcen Gokce**

Turkey

**Dan Gold**

USA

**David Goldblum**

Switzerland

**Johannes Gonnermann**

Germany

**J Gonzalez-Meijome**

Portugal

**Günther Grabner**

Austria

**Enrique Graue-Hernández**

Mexico

**Philip Griffiths**

Gibraltar

**Inna Grishkan**

USA

**Andrzej Grzybowski**

Poland

**Laurent Guilloton**

France

**Priya Gupta**

Canada

**Nigel Hall**

UK

**Inderraj Hanspal**

UK

**Hideaki Hara**

Japan

**Takeshi Harayama**

Japan

**Sveinn Hakon Hardarson**

Iceland

**Andrew Harrison**

USA

**Noriyasu Hashida**

Japan

**Scott Hau**

UK

**Miao He**

China

**Jiucheng He**

USA

**Shikun He**

USA

**Owen Hee**

Singapore

**Steffen Heegaard**

Denmark

**James Hejtmancik**

USA

**Taichi Hikichi**

Japan

**Cornelia Hirn**

Switzerland

**Juha Holopainen**

Finland

**Milton Hom**

USA

**Shigeru Honda**

Japan

**Sadao Hori**

Japan

**Huang Houbin**

China

**Paul Houghtaling**

USA

**Xiufang Hu**

China

**Ruth Huna-Baron**

Israel

**Rahat Husain**

Singapore

**Jeong-Min Hwang**

Korea, South

**Takeshi Ide**

USA

**Michele Iester**

Italy

**Yasushi Ikuno**

Japan

**Makoto Inoue**

Japan

**Jenny Ip**

Australia

**Murat Irkec**

Turkmenistan

**Fakir M Amirul Islam**

Australia

**Domagoj Ivastinovic**

Austria

**Nieraj Jain**

USA

**Tamarra James-Todd**

USA

**Vikram Jayaram**

USA

**Vishal Jhanji**

Hong Kong

**Jian Ji**

China

**Animesh Jindal**

India

**Björn Johansson**

Sweden

**Choun-Ki Joo**

Korea, South

**Jong Hwa Jun**

Korea, South

**Rim Kahloun**

Tunisia

**Freny Kalapesi**

Australia

**Yogita Kanan**

USA

**Costas Karabatsas**

Greece

**Murat Karacorlu**

Turkey

**Dimitrios Karagiannis**

Greece

**Newton Kara-Junior**

Brazil

**Marzieh Katibeh**

Iran

**Kishore Reddy Katikireddy**

USA

**Andreas Katsanos**

Greece

**Sushmita Kaushik**

India

**Srinivasan Kavitha**

India

**Ryo Kawasaki**

Japan

**Stephen Kaye**

UK

**Ramesh Kekunnaya**

India

**Takeshi Kezuka**

Japan

**Jyoti Khadka**

Australia

**Rohit Khanna**

India

**Ahmad Kheirkhah**

USA

**Mee Kum Kim**

Korea, South

**Tae-Im Kim**

Korea, South

**Anthony King**

UK

**James Kirwan**

UK

**Ronald Klein**

USA

**Victoria Knudsen**

USA

**Gergana Kodjebacheva**

USA

**Nageswara Rao Kollu**

USA

**Georgios Kontadakis**

Greece

**Juergen Kopitz**

Germany

**Gabor Koranyi**

Sweden

**Bobby Korn**

USA

**Suheyla Kose**

Turkey

**Parvaiz Koul**

India

**Shingo Koyama**

Japan

**Igor Kozak**

Saudi Arabia

**Vassilios Kozobolis**

Greece

**Florian Kretz**

Germany

**Kaushal Kulkarni**

USA

**Reetu Kundu**

India

**Kazuki Kuniyoshi**

Japan

**Ajay Kuriyan**

USA

**Ingela Lundin Kvalem**

Norway

**Hyung-Woo Kwak**

Korea, South

**Soon Il Kwon**

Korea, South

**Georgios Labiris**

Greece

**Francesco Lacarrubba**

Italy

**Neil Lagali**

Sweden

**Jimmy Lai**

Hong Kong

**Klara Landau**

Switzerland

**Alex Lau**

Singapore

**Augustinus Laude**

Singapore

**Qihua Le**

China

**Chen Le**

China

**Hyun Soo Lee**

Korea, South

**Jiahn-Shing Lee**

Taiwan

**Llewellyn Lee**

Singapore

**Hyung Lee**

Korea, South

**Christopher Seungkyu Lee**

Korea, South

**Jong-Jer Lee**

Taiwan

**Salomé Leibundgut**

Switzerland

**Baihua Li**

UK

**Bin Li**

China

**Qingfeng Liang**

China

**Gerald Liew**

Australia

**K. Sheng Lim**

UK

**Faten Limaiem**

Tunisia

**Chun-Ju Lin**

Taiwan

**Dusheng Lin**

China

**Paloma Liton**

USA

**Bingqian Liu**

China

**Stefan Löfgren**

Sweden

**Federico Luengo Gimeno**

Argentina

**Fiona Luk**

Hong Kong

**Bruno Lumbroso**

Italy

**Ling Luo**

China

**Douglas Lyall**

UK

**Lyubomyr Lytvynchuk**

Ukraine

**Alex Macleod**

UK

**Adriano Magli**

Italy

**George Magrath**

USA

**Sajjad Mahmood**

UK

**Dimitra Makrynioti**

Greece

**Baskaran Mani**

Singapore

**Gianluca Manni**

Italy

**Sonia Manning**

Ireland

**Ahmad Mansour**

Lebanon

**Kaweh Mansouri**

Switzerland

**Srinivas Marmamula**

India

**Michael Marmor**

USA

**Antonio Martinez**

Spain

**Enrico Martini**

Italy

**Leonardo Mastropasqua**

Italy

**Suzana Matayoshi**

Brazil

**Sachin Mathew George**

Singapore

**Hidetaka Matsumoto**

Japan

**Colm Mcalinden**

UK

**Timothy Mcculley**

USA

**Martin Mckibbin**

UK

**Andrew Mcnaught**

UK

**Flavio Medina**

Brazil

**Carmen Mendez-Hernandez**

Spain

**Efstratios Mendrinos**

Greece

**Qianli Meng**

China

**Giuseppe Merla**

Italy

**Carsten H. Meyer**

Switzerland

**Catherine Meyerle**

USA

**Zofia Michalewska**

Poland

**Manuele Michelessi**

Italy

**Giovanni Milano**

Italy

**Helen Mintz-Hittner**

USA

**Ellen Mitchell**

USA

**Masahiro Miura**

Japan

**Sasan Moghimi**

Iran

**Elad Moisseiev**

Israel

**Jiten Morarji**

UK

**Kazuhiko Mori**

Japan

**Naoyuki Morishige**

Japan

**Paolo Morselli**

Italy

**Heather Moss**

USA

**Marlene Moster**

USA

**Eric Muir**

USA

**Tomoaki Murakami**

Japan

**Ann Murchison**

USA

**Ian Murdoch**

UK

**Jenny Myung**

USA

**Kyung-Sun Na**

Korea, South

**Zoltan Nagy**

Hungary

**Vinay Nangia**

India

**Toshio Narimatsu**

Japan

**Piergiorgio Neri**

Italy

**Nuwan Niyadurupola**

UK

**Hiroki Nomoto**

Japan

**Miho Nozaki**

Japan

**Francesco Oddone**

Italy

**Dominik Odrobina**

Poland

**Akio Oishi**

Japan

**Obiekwe Okoye**

Nigeria

**Refik Oltulu**

Turkey

**Oluwole Omolase**

Nigeria

**Cheung-Ter Ong**

Taiwan

**Els Ortibus**

Belgium

**Nicola Orzalesi**

Italy

**Andrew Osborne**

UK

**Eoin O'Sullivan**

UK

**Atsushi Otani**

Japan

**Yoko Ozawa**

Japan

**Joseph Panarelli**

USA

**Georgios Panos**

Switzerland

**Eleni Papageorgiou**

UK

**Vasileios Papaliagkas**

Greece

**Sang-Woo Park**

Korea, South

**Ki Ho Park**

Korea, South

**Hae-Young Park**

Korea, South

**Annette Parkinson**

UK

**Mrinali Patel**

USA

**Niall Patton**

UK

**Mikk Pauklin**

Estonia

**Carlos Pavesio**

UK

**Gökhan Pekel**

Turkey

**Xijia Peng**

China

**Henry Perry**

USA

**George Peters**

USA

**Sergio Petroni**

Italy

**Ioannis N. Petropoulos**

UK

**Francesco Pichi**

Italy

**Antonio Pinna**

Italy

**Susanne Pitz**

Germany

**Guillermo Plaza**

Spain

**Pawan Prasher**

India

**Frank Proudlock**

UK

**Malgorzata Przybylo**

Poland

**Renata Puertas**

UK

**Xuejiao Qin**

China

**Guoping Qing**

China

**Yi Qu**

China

**Luciano Quaranta**

Italy

**Rajesh Rajagopalan**

Singapore

**Raju Rajala**

USA

**Thiagarajan Raman**

India

**Manuel Ramirez**

Mexico

**Pradeep Ramulu**

USA

**Mohammad Reza Razeghinejad**

Iran

**Andri Riau**

Singapore

**Joao Crispim Ribeiro**

Brazil

**Gabriella Ricciardelli**

Italy

**Ivano Riva**

Italy

**Dana Robaei**

Australia

**Gloria Roberti**

Italy

**Alan Robin**

USA

**Murilo Roggia**

Japan

**Peter Rolfe**

Italy

**Teresa Rolle**

Italy

**Shisong Rong**

China

**James Rosenbaum**

USA

**Luca Rossetti**

Italy

**Sylvain Roy**

Switzerland

**Eliana Rulli**

Italy

**Mohammad Sadiq**

USA

**Osamah Saeedi**

USA

**Wataru Saito**

Japan

**Tsutomu Sakai**

Japan

**Daniel Salchow**

Germany

**Maria Letizia Salvetat**

Italy

**Roshini Sanders**

UK

**Tatsuhiko Sato**

USA

**Simon Saule**

France

**Robert W Sault**

Canada

**Judy Savige**

Australia

**Giacomo Savini**

Italy

**Nicoline Schalij-Delfos**

Netherlands

**Patrik Schatz**

Sweden

**Victor Schepkin**

USA

**Katrina Schmid**

Australia

**Dieter Schmidt**

Germany

**Lynn Schoenfield**

USA

**Edina Schweighoffer**

UK

**Michael Seider**

USA

**Walter Sekundo**

Germany

**Jeremiah Seni**

Tanzania

**Parag Shah**

India

**Syed Shah**

USA

**Rupal Shah**

India

**Mahesh Shanmugam P**

India

**Shiwani Sharma**

Australia

**Ismail Shatriah**

Malaysia

**Yasmin Shayesteh**

USA

**Kei Shinoda**

Japan

**Chieko Shiragami**

Japan

**Stephen Shumack**

Australia

**Isabel Signes-Soler**

Spain

**Eduardo Silva**

Chile

**Huibert Jan Simonsz**

Netherlands

**Trefford Simpson**

Canada

**Martin Sin**

Czech Republic

**Michael Singer**

USA

**Rishi Singh**

USA

**Eric Singman**

USA

**Simon Skalicky**

UK

**Katarzyna Skonieczna**

Poland

**Frank Sloan**

USA

**Andrew Fraser Smith**

Canada

**Wendy M Smith**

USA

**Kamal Solaiman**

Egypt

**Peter Soliz**

USA

**Gianluca Sottilotta**

Italy

**Alex Spratt**

UK

**Oliver Stachs**

Germany

**Philip Stanley**

Singapore

**Johannes Steinberg**

Germany

**Douglas Stewart**

USA

**Irene Stratton**

UK

**Rupert Wolfgang Strauss**

USA

**Tobias Stupp**

Germany

**Tatiana Suarez**

Spain

**Ahalya Subramanian**

UK

**Prem Sagar Subramanian**

USA

**Young-Woo Suh**

Korea, South

**Rufino Suilva**

Portugal

**Michelle Sun**

Australia

**Hisaharu Suzuki**

Japan

**Takashi Suzuki**

Japan

**Meenakshi Swaminathan**

India

**Hiroki Takada**

Japan

**Johnson Tan**

Singapore

**Petrina Tan**

Singapore

**Suphi Taneri**

Germany

**Keiji Tanese**

Japan

**Shibo Tang**

China

**Masaki Tanito**

Japan

**Kristina Tarczy-Hornoch**

USA

**Simon Taylor**

UK

**Umit Y Tekelioglu**

Turkey

**Alejandro Tello**

Colombia

**Stephen Teoh**

Singapore

**Preethi Thiagarajan**

USA

**Margarita Todorova**

Switzerland

**Minoru Tomita**

Japan

**Paul Tomlins**

UK

**Marc Töteberg-Harms**

Switzerland

**Lisa Toto**

Italy

**Luis Trujillo**

USA

**Chieh-Chih Tsai**

Taiwan

**Rong Kung Tsai**

Taiwan

**Michael Tsatsos**

UK

**George Turner**

UK

**Addepalli Uday**

India

**Takashi Ueta**

Japan

**Marta Ugarte**

UK

**Canan Utine**

Turkey

**Monika Valtink**

Germany

**Suzanne Van Landingham**

USA

**Abhay Vasavada**

India

**Clemens Vass**

Austria

**Edbhergue Ventura Lola Costa**

Brazil

**Nitin Verma**

Australia

**Monica Vetter**

USA

**Lingam Vijaya**

India

**Stephen Vincent**

Australia

**Francesco Viola**

Italy

**Gianni Virgili**

Italy

**Jan Albert Vos**

Netherlands

**Brendan Vote**

Australia

**Katrin Wacker**

Germany

**Nan-Kai Wang**

Taiwan

**Mengmeng Wang**

China

**Kai Jie Wang**

China

**Naushin Waseem**

UK

**Heather Waterman**

UK

**Alfred Wegener**

Germany

**Shihui Wei**

China

**Andreas Weinberger**

Germany

**Rong Wen**

USA

**Feng Wen**

China

**Adam Wenick**

USA

**Helmut Wilhelm**

Germany

**Mark Willcox**

Australia

**Geraint Williams**

UK

**Pete Williams**

USA

**Gabriel Willmann**

Germany

**Kate Windridge**

UK

**Yulia Wolfson**

USA

**Se Joon Woo**

Korea, South

**Emily Woodman**

Australia

**Wei-Chi Wu**

Taiwan

**Jason Yam**

Hong Kong

**Eylem Yaman Pinarci**

Turkey

**Morgan Yang**

Singapore

**Yan-Ning Yang**

China

**Paul Yang**

USA

**Yit Yang**

UK

**Tun K Yeo**

Singapore

**Gui-Shuang Ying**

USA

**Shao Onn Yong**

Singapore

**David Yoo**

USA

**Young Hee Yoon**

Korea, South

**Jing Yu**

China

**Shanshan Yu**

China

**Young Suk Yu**

Korea, South

**Tomasz Zarnowski**

Poland

**Hajo Zeeb**

Germany

**Xiongze Zhang**

China

**Sen Zhang**

China

**Zuoming Zhang**

China

**Yingfeng Zheng**

China

**Hua Zhong**

China

